# Neonatal rhizomelic chondrodysplasia punctata type 2 caused by a novel homozygous variant in the *GNPAT* gene

**DOI:** 10.1002/ccr3.7504

**Published:** 2023-06-13

**Authors:** Jamal Sayed, Ahmed Gamal, Abdulrahman Theyab, Mohamed Algahtani, Banan Bakheet Aldaadi

**Affiliations:** ^1^ Security Forces Hospital Makkah (SFHM) Makah Saudi Arabia; ^2^ College of Medicine Al‐Faisal University Riyadh Saudi Arabia; ^3^ Department of Laboratory and Blood Bank Security Forces Hospital Makkah Makah Saudi Arabia; ^4^ Department of Radiology Security Forces Hospital Makkah Makah Saudi Arabia

**Keywords:** genetic testing, *GNPAT* gene, mutation, neonatal RCDP, RCDP

## Abstract

Rhizomelic chondrodysplasia punctata (RCDP) is a rare disorder (~1 in 100,000 live births) of faulty plasmalogen biosynthesis and defective peroxisomal metabolism. RCDP type 2 is specifically caused by glyceronephosphate O‐acyltransferase (*GNPAT*) gene mutations and is inherited as an autosomal recessive trait. The disorder is characterized by skeletal abnormalities, distinctive facial features, intellectual disability, and respiratory distress. The case report describes a newborn baby with a dysmorphic facial appearance and skeletal abnormalities who was admitted to neonatal intensive care with respiratory distress. His parents were first cousins. The whole exome sequencing for this patient identified an interesting homozygous variant in the *GNPAT* gene [GNPAT (NM_014236.4):c.1602+1G>A (p.?), Chr1 (GRCh37):g.231408138G>A]. This case report aims to highlight the patient's clinical presentation with the variant and the whole exome sequencing, indicating the identification of a novel mutation in the *GNPAT* gene causing RCDP type 2.

## 1 INTRODUCTION

Rhizomelic chondrodysplasia punctata (RCDP) is an uncommon inherited disease caused by an impaired ability of plasmalogen synthesis. Plasmalogens are vinyl ether that contains membrane phospholipids. These are essential to maintain the cellular membrane's structure and function properly. The pathology of RCDP is associated with mutations in five genes involved with plasmalogen biosynthesis.^1^ RCDP type 2 (OMIM 222765) is an autosomal recessive disorder that arises secondary to mutations in the acyl‐CoA: dihydroxyacetone phosphate acyltransferase (*DHAPAT*) gene.^1^ RCDP children have medical complications, and during their early childhood or infancy, many have died. Most patients who endure beyond the neonatal stage have serious physical disabilities and impaired cognition.^2^ Shortening of the proximal limb, mental retardation, and severely disturbed endochondral bone formation are the symptoms of RCDP.^3^, ^4^ Proximal long bones shortening, punctate calcifications in the metaphysis and epiphysis of long bones, and the thoracic and lumbar vertebrae, facial dysmorphism with a prominent forehead, broad nasal bridge, hypertelorism, epicanthus, micrognathia, and high arched palate are the main features of the disease. Almost all affected individuals have cataracts visible at birth or developing during early childhood, with severe intellectual disability and growth retardation.^5^ RCDP type 2 affects less than one in 100,000 people globally. This article aims to present a case with a novel splicing homozygous variant in the glyceronephosphate O‐acyltransferase (*GNPAT*) gene, which has not been reported before in the literature, with distinct clinical features which describe RCDP type 2.

## 2 CASE REPORT

The late premature baby was admitted to neonatal intensive care after birth due to respiratory distress and dysmorphic facial appearance, and short limbs. The mother was 26 years old and in good health, and the father was 31 years old with first‐degree consanguinity and gave birth to a male baby at 36 weeks. During pregnancy, prenatal care was provided to the mother. She had no chronic diseases and had never been exposed to known teratogenic agents. Her prenatal ultrasonography assessments at 35 weeks gestation depict fetal growth below the fifth percentile, which is significant. All short and long bones, such as the radius, ulna, femur, tibia, and fibula lengths were below the reference percentiles for the gestational age, with the following measurement: [tibia is 51 mm, which corresponds to 31.2 weeks, the fibula is 51 mm corresponding to 30.5 weeks, the humerus is 38 mm corresponding to 23.1 weeks, the radius is 44 mm corresponding to 33.3 weeks, ulna is 51 mm corresponding to 32.2 weeks]. The mother had a history of first‐trimester spontaneous abortion and one preterm delivery at 26 weeks. She died after a few days in the neonatal intensive care unit due to severe respiratory distress and neonatal early‐onset sepsis. She had gestational diabetes, which was controlled on a diet. The physical examination revealed the weight at birth was 2205 g (<25th percentile for gestational age, which is equal to −0.675 standard deviations), the length was 45 cm (25th percentile, which equals to −0.675 standard deviations), with a head circumference of 31 cm (<25th percentile, which equals to −0.675 standard deviations). The ratio of the upper to the lower segment was 1.6:1. There was a proximal shortening of the upper and lower limbs in the baby. Other dysmorphic features included a depressed nasal bridge, broad nose, coarse facial features, and long philtrum. The baby had multiple contractures at the thigh, knee, shoulder, elbow, wrist, and fingers (arthrogryposis multiplex congenita). Other systemic examinations, besides evidence of restrictive lung disease, make the baby oxygen‐dependent on maintaining his normal oxygenation. The ophthalmological examination showed a bilateral polar cataract. The radiological finding of the patient showed finding compatible with the diagnosis of RCDP.

**FIGURE 1:**
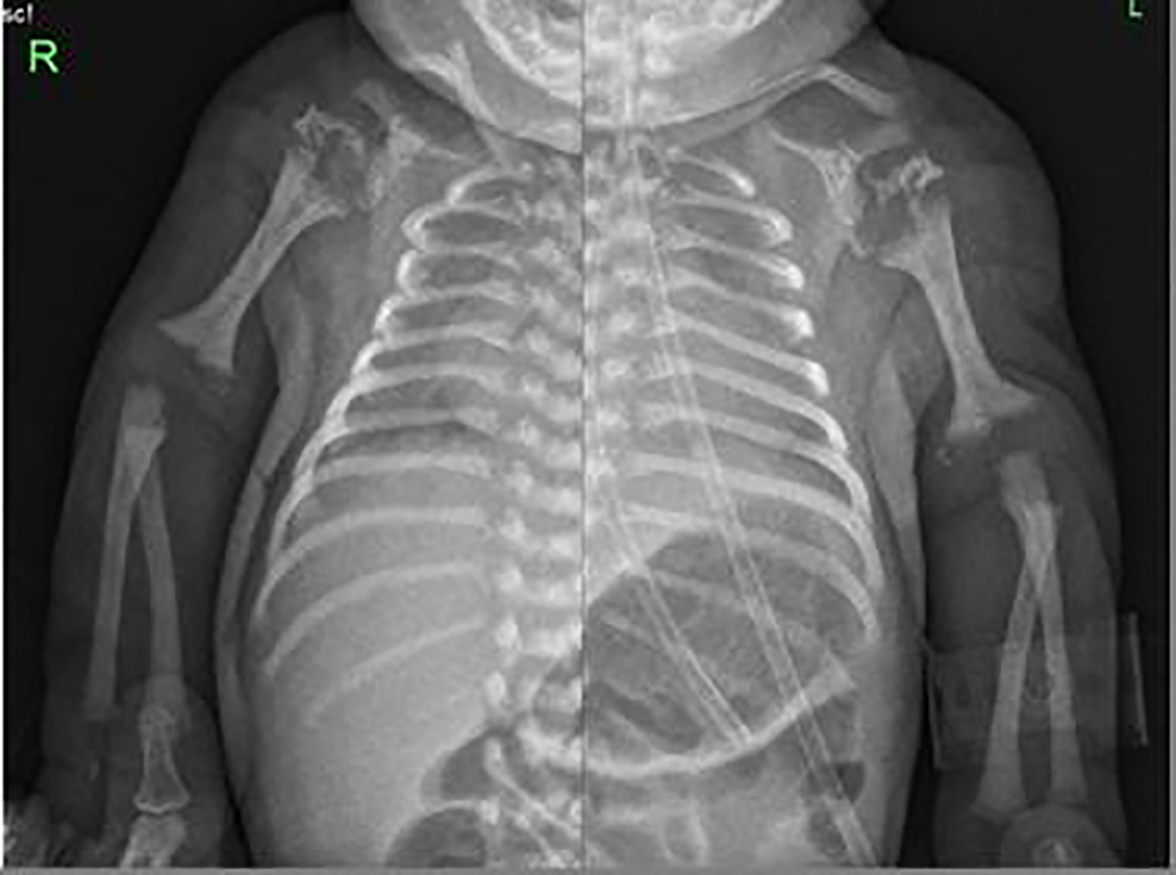
Upper limbs radiographs show rhizomelic shortening of both humeri, flared metaphyses, and stippled calcifications of proximal and distal epiphyses.

**FIGURE 2:**
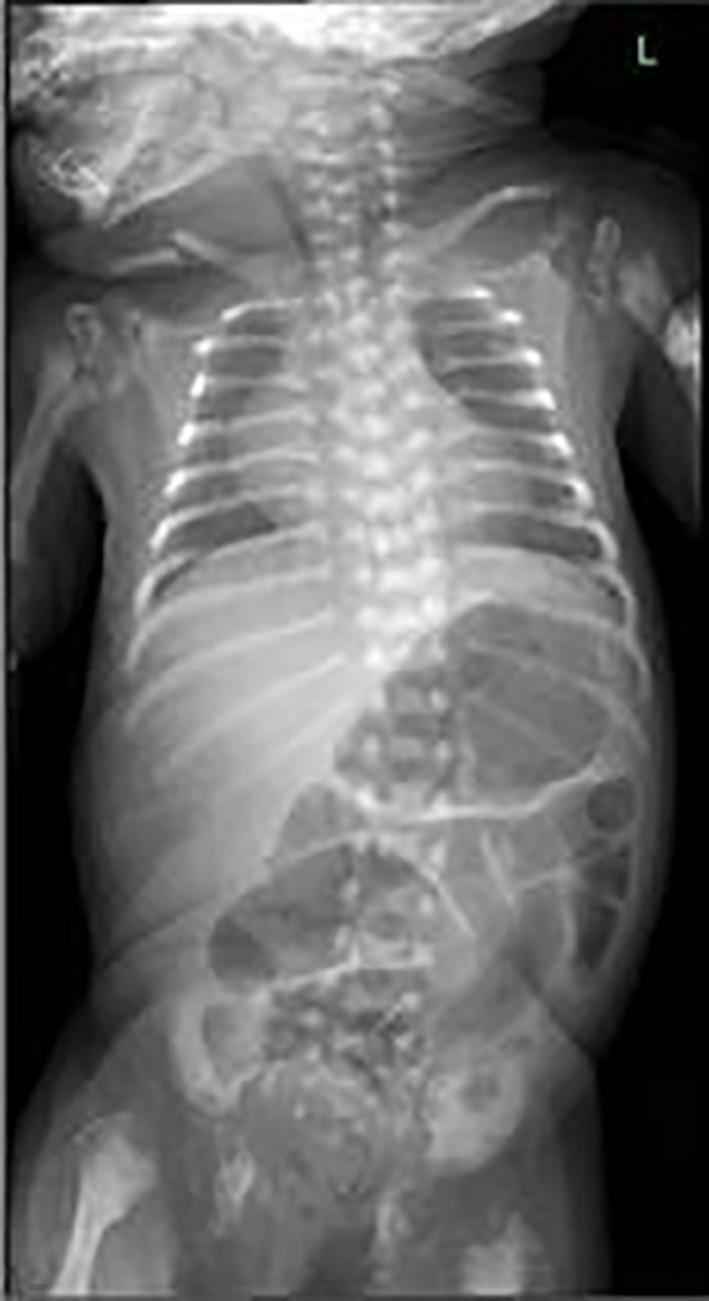
Chest and abdomen radiographs shows extensive punctate vertebral calcification.

**FIGURE 3:**
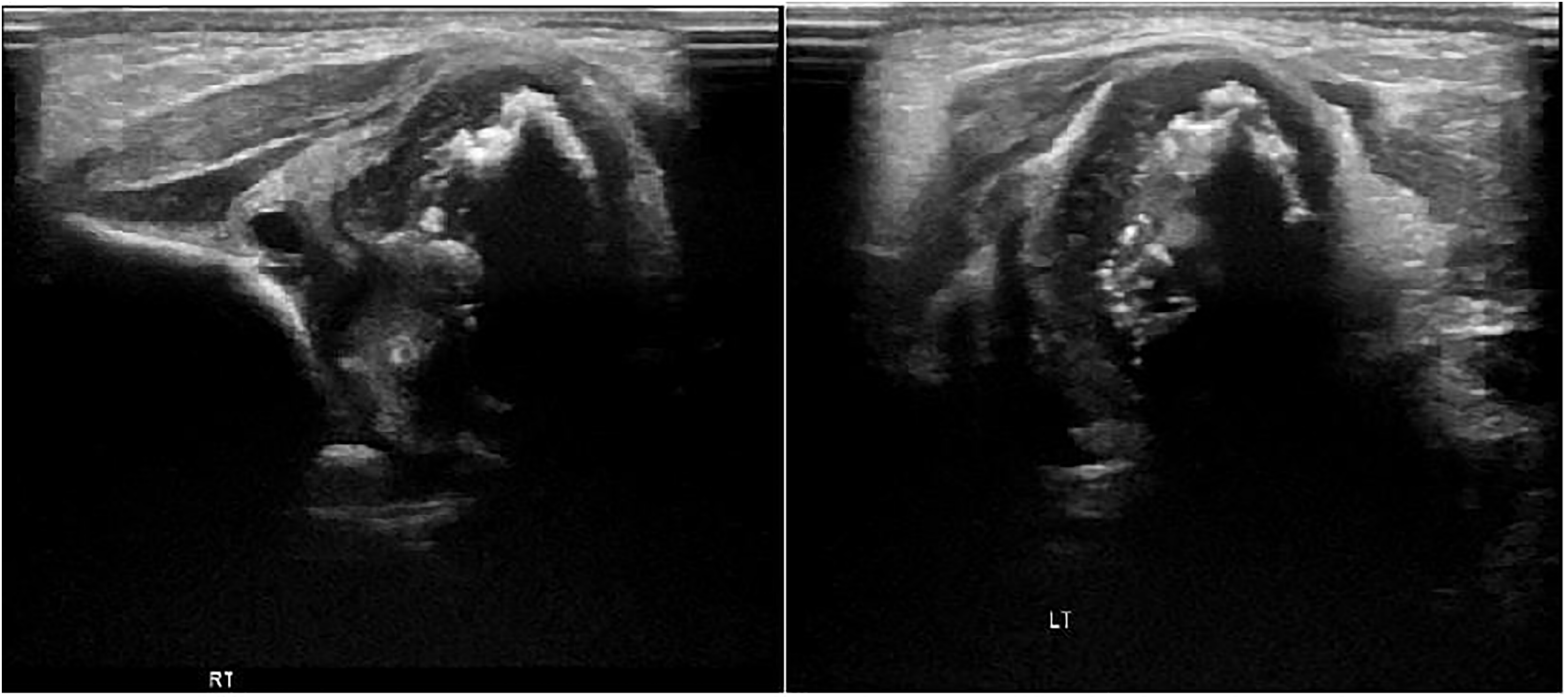
Ultrasound image of both femoral heads shows stippling calcification of both femoral epiphyses.

Laboratory findings:

1. A skeletal examination revealed symmetry in bilateral shortening of the humerus and femur with punctate epiphysis due to stippled calcification (Figures 1 and 2), and diaphyseal thickening with metaphyseal splaying and fraying were noted. Bilateral acetabula erosion was present with decreased lung volumes.
2. Hip ultrasound (US) showed an abnormal configuration of both hip joints involving their opposing bony and cartilaginous components with multiple stippled echogenic foci and hypertrophied cartilage. This results in the displacement of both femoral epiphyses away from their articulating acetabulum (Figure 3). Complete blood count (CBC), abdominal ultrasonography, biochemical parameters, and cranial ultrasonography were normal. The cervical vertebrae, nasopharynx, and neck CT results were normal.
3. Echocardiography showed a small ventricular septal defect (VSD) of 2 mm bidirectional shunt, a small atrial septal defect (ASD) of 2–5 mm Lt to Rt shunt, and good cardiac function.

**FIGURE 4:**
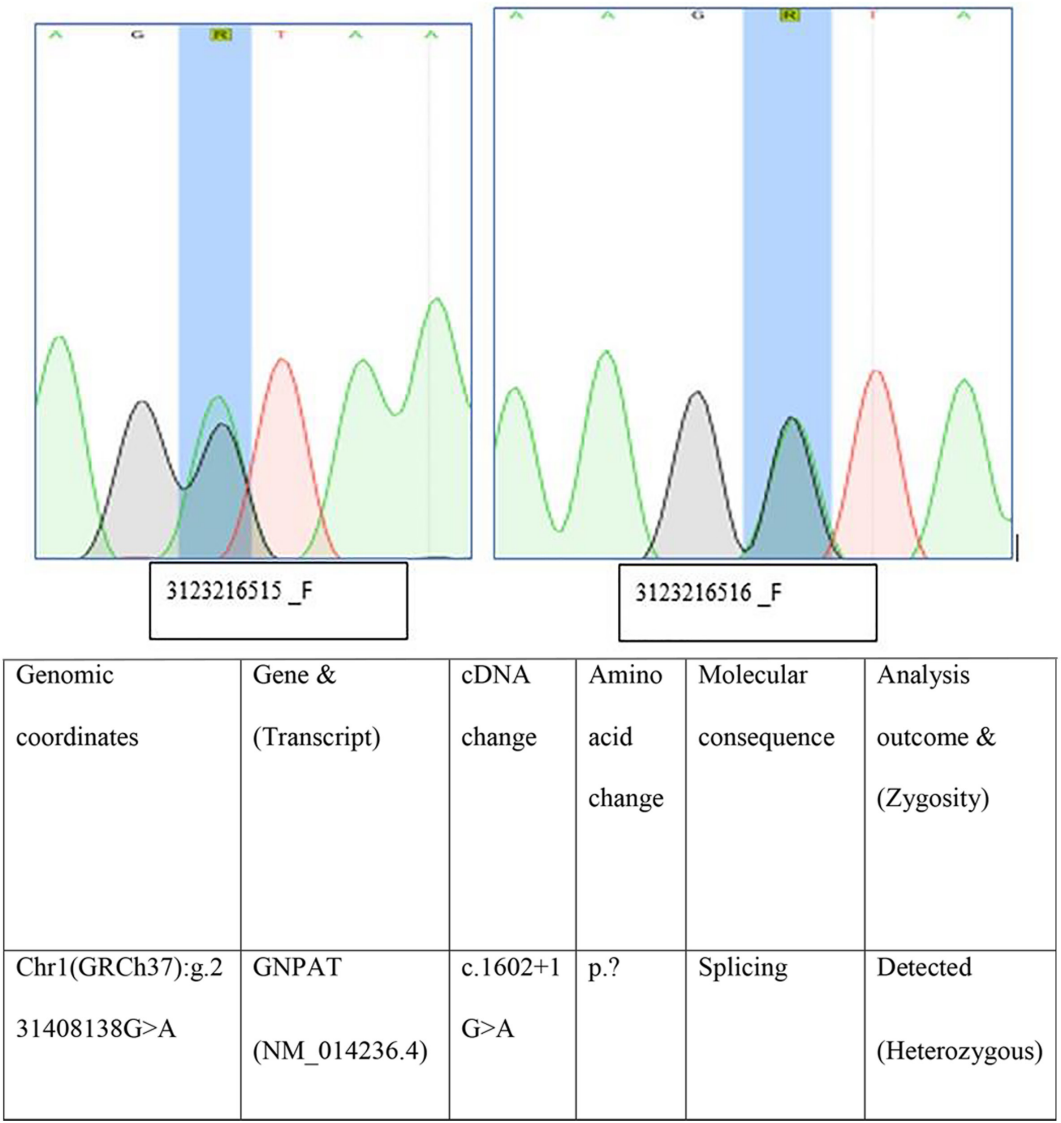
Sanger sequencing of parent.

The whole exome sequencing for this patient identified a novel splicing homozygous variant. GNPAT (NM_014236.4):c.1602+1G>A (p.?), Chr1 (GRCh37):g.231408138G>A. There has never been a literature report on this variant, and neither has been found in general population databases (gnomAD: no frequency). A donor splice site is affected by this sequence change in the *GNPAT* gene's intron 11 positions. According to the algorithms developed to predict the effects of sequence changes on RNA splicing, the consensus splice site may get disrupted by this variant, but published transcriptional studies have not confirmed this prediction. Variations in donor and acceptor splice sites result in loss of protein function (PMID: 16199547), and loss‐of‐function variants in the *GNPAT* gene are pathogenic (PMID: 9536089, 21990100). This variant has been designated as potentially pathogenic based on this information and ACMG guidelines compatible with the phenotype. Pathogenic variants in *GNPAT* gene RCDP type 2. The peroxisomal disorder RCDP type 2 is characterized by disproportionately short stature. It primarily affects the proximal parts of the extremities. It includes typical facial appearance, a broad nasal bridge, epicanthus, high‐arched palate, dysplastic external ears, micrognathia, congenital contractures, characteristic ocular involvement, dwarfism, and severe mental retardation with spasticity. Considering the homozygous pathogenic variant in the *GNPAT* gene and the supportive phenotype of the patient, a genetic diagnosis of RCDP type 2 is confirmed. The homozygous splicing variant effect on protein structure needs an RNA function study. This study was not done in our case as the family refuses to go for further study, and his variant is classified as pathogenic based on ACMG guidelines and compatible with the phenotype we appreciate is the limitation of our case report. The diagnosis was informed to the parents, genetic counseling was given. Sanger sequencing was done for both parents, confirming a heterozygous carrier for this variant. The graph explains this for both samples (Figure 4). The case is now 4 months old, feeding through a nasogastric tube, weighing 3.450 g with slow growth and difficulty gaining weight at home care with oxygen support of 28% by nasal cannula. His cardiac lesion is static with no cardiac medication; cataract surgery was also planned with an ophthalmologist.

## 3 DISCUSSION

Chondrodysplasia punctata (CDP) is a clinical and genetic condition of heterogeneous dysplasia. One out of every 100,000 live births has stippled, punctate calcifications around joints and inside the cartilages.^6^, ^7^ Rhizomelic type chondrodysplasia punctata (RCDP), linked to peroxisomal enzyme disorder, is inherited in an autosomal recessive manner. The past literature classifies RCDPs clinically into five types, which are all autosomal recessive. The associated genes are as follows: RCDP type 1 with *PEX7*, RCDP type 2 with *GNPAT*, RCDP type 3 with *AGPS*, RCDP type 4 with *FAR1*, and RCDP type 5 with *PEX5*.^8^ Therefore, the *GNPAT* gene is our focus, as this gene is associated with plasmalogen synthesis. To demonstrate *GNPAT* encodes a peroxisome‐specific enzyme, the acyl‐CoA: glyceronephosphate O‐acyltransferase (DHAPAT), which is only located within peroxisomes and is responsible for intraperoxisomal acylation of DHAP to acylDHAP thus, the enzyme is responsible for the function of acyltransferase in ether‐phospholipid synthesis which is irreplaceable by microsomal or mitochondrial acyltransferases signifying the *GNPAT* role in plasmalogen synthesis.

This case report discusses a late premature baby with respiratory distress, dysmorphic facial appearance, and short limbs. Skeletal dysplasia (SD) was anticipated with perinatal ultrasound, which showed shortening of all limbs despite a negative family history of SD. The growth parameters showed below the fifth percentile. The postnatal assessment described distinct facial features with striking proximal shortening of upper and lower limbs. His skeletal survey showed discrete punctate cartilaginous calcifications and a short proximal skeleton, likely CDP. The dysmorphic facial features were also noted in the earlier report of a 16‐day‐old baby girl from Saudi Arabia, where she had low set ears, long philtrum, and upslanting palpebral fissures of both eyes.^9^ Our case report had similar findings with a depressed nasal bridge, broad nose, coarse facial features, and long philtrum. The baby had multiple contractures at the thigh, knee, shoulder, elbow, wrist, and fingers (arthrogryposis multiplex congenita). A bilateral symmetry in the shortening of the humerus and femur with punctate epiphysis due to stippled calcification was observed, and diaphyseal thickening with metaphyseal splaying and fraying was noted. There was a previously reported case of RCDP type 3 with a novel AGPS genetic variant, where punctate calcifications were seen in the proximal humerus, proximal femur, and upper spine.^9^ His differential diagnosis includes genetically inherited CDPs, with maternal autoimmune disease, exposure to warfarin, and fetal alcohol syndrome. The mother tested negative for autoimmune disease in the lab. In addition, no prior history of drug or alcohol use or any symptoms was observed.^10^ A bilateral polar cataract was observed in this case, comparable to an earlier reported case with congenital cataracts in three reported patients.^9^ The disease was explained to the parents whose children were provided genetic counseling. The patient went home on oxygen support with a home care service program and multidisciplinary outpatient follow‐up.

## 4 CONCLUSION

Rhizomelic chondrodysplasia punctate type 2 is an ultra‐rare disease with skeletal manifestations and biochemical abnormality in the form of low plasminogen levels. Our case further supports this disease severity with a novel splicing homozygous variant, which has not been reported before in the literature.

## AUTHOR CONTRIBUTIONS

**Jamal Sayed:** Conceptualization; data curation; writing – original draft; writing – review and editing. **Ahmed Gamal:** Data curation; writing – original draft; writing – review and editing. **Abdulrahman Theyab:** Data curation; writing – original draft; writing – review and editing. **Mohamed Algahtani:** Data curation; investigation; writing – original draft; writing – review and editing. **Banan Bakheet Aldaadi:** Data curation; formal analysis; investigation.

## FUNDING INFORMATION

Funding by authers.

## CONFLICT OF INTEREST STATEMENT

The authors have no conflict of interest to declare.

## ETHICS STATEMENT

The manuscript has been reviewed and approved by the IRB and Public Affairs Office.

## CONSENT

The authors have confirmed that patient consent has been signed and collected in accordance with the journal's patient consent policy.

## Data Availability

Not applicable.
